# Brain Performance *versus* Phase Transitions

**DOI:** 10.1038/srep12216

**Published:** 2015-07-20

**Authors:** Joaquín J. Torres, J. Marro

**Affiliations:** 1 Institute Carlos I for Theoretical and Computational Physics, Granada, E-18071, Spain

## Abstract

We here illustrate how a well-founded study of the brain may originate in assuming analogies with phase-transition phenomena. Analyzing to what extent a weak signal endures in noisy environments, we identify the underlying mechanisms, and it results a description of how the excitability associated to (non-equilibrium) phase changes and criticality optimizes the processing of the signal. Our setting is a network of *integrate-and-fire* nodes in which connections are heterogeneous with rapid time-varying intensities mimicking *fatigue* and *potentiation*. Emergence then becomes quite robust against wiring topology modification—in fact, we considered from a fully connected network to the Homo sapiens connectome—showing the essential role of synaptic flickering on computations. We also suggest how to experimentally disclose significant changes during actual brain operation.

Following previous indications^1^,^2^, a wide variety of experiments and computer studies have recently described features of brain functions and structure in terms of phase transitions and criticality^3^,^4^,^5^,^6^,^7^,^8^,^9^,^10^,^11^,^12^,^13^,^14^,^15^,^16^,^17^,^18^,^19^,^20^,^21^,^22^,^23^,^24^,^25^,^26^,^27^,^28^,^29^. These two concepts, particularly when they concern non-equilibrium conditions and complex networks, as it is the case, involve a diverse and even bizarre phenomenology and, all at once, a rather well-defined setting which offers powerful methods of analysis^30^,^31^,^32^,^33^,^34^. Deepening on such analogy is therefore important.

The many indications that the phase-transition concept is pertinent to better understanding the brain have now firm theoretical support. In particular, the claim that long ranged correlations and avalanches of many different sizes take place in the brain has became quite plausible. In fact, the intricate networking of the nervous system and the associated lack of both linearity and thermodynamic equilibrium implies an underlying essential heterogeneity which prompts the combination of scales along broad spatial and time ranges thus inducing a type of spontaneous critical behavior^27^,^35^,^36^. Also relevant within this context is the recognition that criticality induced by quenched disorder^37^ may occur in these systems. Furthermore, synaptic complexity is known to favour kind of roaming dynamics which is irregular as in the neighborhood of a critical point or at the *edge of chaos*^13^,^38^,^39^,^40^,^41^ and information theory indicates that looking for functional advantages in dealing with diverse environmental conditions might have driven living systems to such critical conditions^29^.

Locating in practice a phase transition—and, therefore, uncovering such important phenomenology, i.e., all those facts of great potential scientific relevance—which may be easy in a mathematical model^30^,^42^, is generally a difficult task in biological systems, however^10^,^28^. In this paper, by means of computer simulations, we first present a coherent description of a family of intriguing conditions or “phases” , and of the transitions between them. The resulting whole picture happens to be most appealing to be correlated with brain behavior, as it has already been done so to some extent, as noticed above. In a natural way we then come to outline a simple experimental method which, based on how weak signals are sometimes able to outlast high levels of noise, may serve to uncover the occurrence of phase transitions and criticality during actual brain operation. The present study thus strengthens, and will help to deep on the understanding of the mentioned widely-held analogy. An important side result of our work is that it makes quite evident how the power and versatility of the brain mainly relies on the dynamic complexity of synapses.

## Model

Our point may already be perfectly illustrated with the following simple model. Consider a network of *N* linked *integrate-and-fire* neurons^43^, that is, each node, *i* = 1,…..*N* is described by a positive-defined potential which evolves in time according to
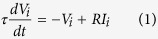
with a cutoff, *V*_*i*_ ≤ *V*_cut_, where *τ* and *R* are characteristic time and resistance constants. This is more realistic *a priori* than assuming binary neurons, *s*_*i*_ = 1, 0, but one may obtain this case from the other by simply taking *s*_*i*_ = 1 while *V*_*i*_ is at its maximum or exceeds the cutoff and *s*_*i*_ = 0 otherwise. The input current will in general have several sources, namely,
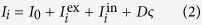
where *I*_0_ is a characteristic constant which sets the range of interest, 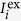 represents the input, that is, an arbitrary, generally weak external signal to be determined, 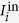 stands for the sum of synaptic currents arriving to neuron *i* from all its neighbors *j* and *ς* is a Gaussian noise of zero mean and unity variance. *D* is thus a main parameter intended to describe the total intensity of a sum of intrinsic uncorrelated signals (e.g.) from other areas of the brain or some extra noise or a combination of both.

We shall further assume that synapsis are dynamic^44^,^45^,^46^,^47^. More specifically,
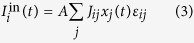
where *A* is a normalization constant and the coefficients *J*_*ij*_ are synaptic weights according to the Hebbian prescription,
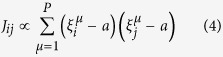
which involves *P* given patterns each consisting of *N* binary variables, 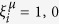. Eq. (4) is the familiar assumption except that we account for the expected lack of symmetry of “memories” in nature, and make explicit the measure of asymmetry 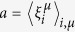 in the considered set. The factor *x*_*j*_(*t*) in eq. (3), which is expected to have only a relevant dependence on the presynaptic neuron^48^, accounts for rapid time changes of the synaptic weights that have been reported as due to intense and repeated use of the involved connection. *ε*_*ij*_ is the adjacency matrix describing the structure of the network. In order to isolate the network influence, we first studied in most detail a fully connected network; however, other cases have also considered and our point as described above happens to be rather independent of the network structure.

**Figure 1:**
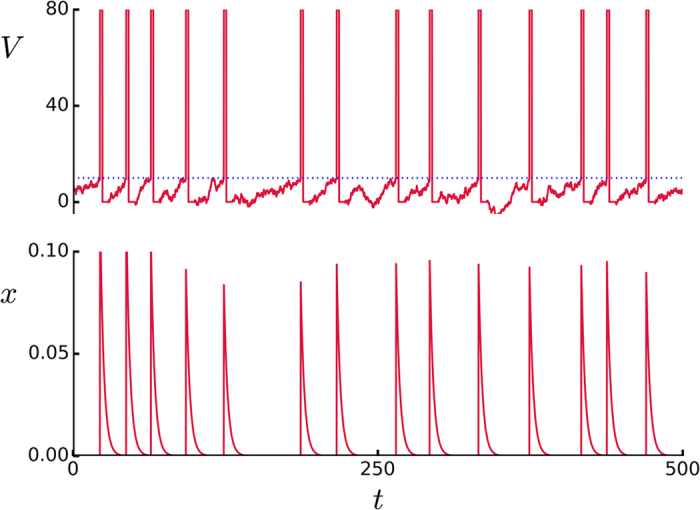
Typical model variations with time (ms) of any V_i_ (mV) and of the corresponding x_i_ as given by the equations^46^: 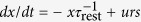
, 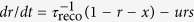 and 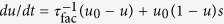, where *s *= *s* (*t*) is always zero but unity when V reaches the cutoff V_cut_ (horizontal dotted line in the top graph) as defined in the main text.

A simple though still relevant choice consists in assuming^13^,^19^ that neurons are binary units with stochastic dynamics and that *x* in eq. (3) is a random variable, e.g., having a bimodal distribution which represents the possibility that the corresponding synaptic intensity is either weakened (*depression*) or reinforced (*facilitation*) in an amount which depends on the degree of order at that time. More elaborated is assuming, as we shall do here following the original description^46^, that *x* is a function of *V*. Specifically, as described in the caption of Fig. 1, it is assumed that *x* and two other variables control the fraction of different types of neurotransmitters, which results into the three indicated coupled differential equations with time parameters that characterize the relevant processes. The constants mentioned in Fig. 1 are set, as in the rest of the paper unless otherwise indicated, *τ*_reco_ = 300 ms, *τ*_rest_ = 3 ms, *τ*_fac_ = 50 ms, *A* = 45 pA, and *R* = 0.1 GΩ; *u*_0_ = 0.2 in Fig. 1 and 0.5 in the rest of the paper. The parameter values here are set according to familiar biological considerations^46^,^49^ and, eventually, based on simplicity considerations. For example, |*J*_*ij*_| happens to be too small in practice, and it needs to be renormalized with a factor (that we set 10^3^) to ensure a significant effect. Also, we used the mean connectivity degree instead of *N* in the denominator of *J*_*ij*_, so that we have comparable mean synaptic current per neuron for different choices of the network matrix *ε*_*ij*_.

The picture of interest can be made explicit by monitoring a few magnitudes. One is the *mean net activity*,
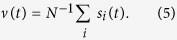
This is a measure of the experimentally observable *mean firing rate* of a neuron (in the case of a recurrent network as it is the case here). Assuming stimuli 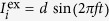 of small amplitude, we shall be concerned on how they behave under *noise Dς* (more realistic is perhaps the case of a stimulus consisting in a spike train, and we have also studied the effect of inhomogeneous Poisson spike trains with mean firing rate *λ*_0_[1+*b*sin (2π*ft*)] where *λ*_0_ and *b* are positive constants. However, no significant new facts thus emerge and the sinus signal is sometimes more convenient to visually follow its influence on the response). Therefore, we are interested on the relative intrinsic variations of the input/output correlation. This is monitored here via the function 
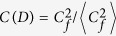
, where *C*_*f*_ is the Fourier coefficient of *v*(*t*) for the frequency of the stimulus. We checked that our results are very clearly observed for any *f* between 0.75 and 3Hz, sharper the lower *f* is, and we thus set *f* = 1.5 Hz throughout this paper. The average 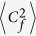 in the denominator of this definition, intended to wash out unimportant fluctuations, is over a small range of frequencies around but excluding the stimulus value *f.* Note that *C*(*D*) may be viewed as our “order parameter” which will help to clearly distinguish between different types of order or “phases”. In addition, as an intuitive characterization of the type of order which is developing, we also monitored the time evolution of the similarity between the current state of the system and each of the involved patterns as measured by the corresponding *overlaps* defined as
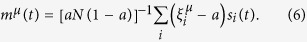


Our main results happen to be quite robust against modifications of model details—e.g., they essentially hold for the relatively simpler models in^13^,^19^—so that we shall first be concerned with the simplest case in this section, and then make some references to other cases in our final discussion.

## Emergent phases and crossovers

The above biologically-motivated setting exhibits two principal features to be mainly associated with the short-time mechanisms of synaptic depression and facilitation. One is that the system behaves as an *excitable medium*^9^,^13^ and, consequently, a weak signal is able to coexist with substantial ambient noise. In a sense, the signal propagates undamped trough the system, given that it may be detected at any site or region, unlike the familiar case of sound transmission in the air. Those mechanisms also originate heterogeneous order at the mesoscopic and macroscopic levels resulting in (non-equilibrium) order and phase transitions. And most remarkable is that these two features, namely, development of order and good propagation of stimuli competing with noise, are closely related to each other. To complete the picture, this phenomenology adds up to, and destabilizes in practice the convergence towards attractors that is known to be induced by the Hebbian long-time plasticity^50^.

**Figure 2:**
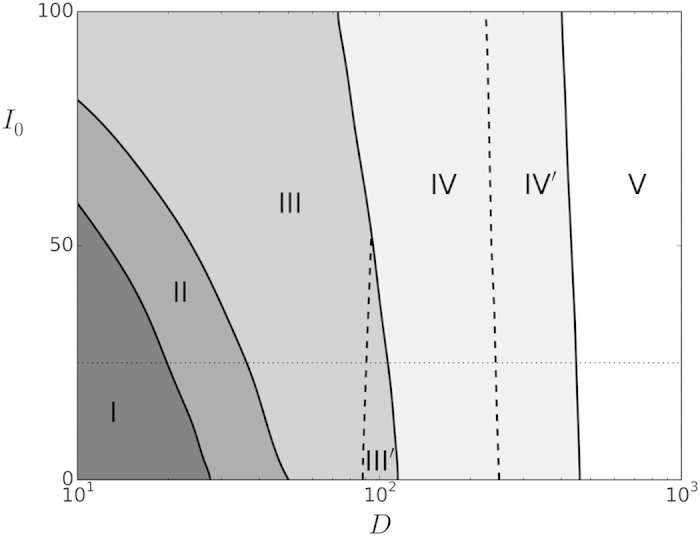
Schematic phase diagram showing *I*_0_ against *D* both in pA units, delimiting the different conditions the model exhibits during computer simulations when *d *= 0 (no signal) as the noise parameter *D* is (logarithmically) increased from left to right. This mainly follows from analysis of the maximum overlaps (see Fig. 6). The dotted horizontal line marks *I*_0_ = 25 pA to which Figs 3, 4, 5, 6 refer. The solid and dashed lines indicate phase changes as discussed in the main text. Here, *τ*_reco_ = 300 ms and *τ*_fac_ = 0 so that the system accounts for synaptic depression but not for reinforcement.

**Figure 3:**
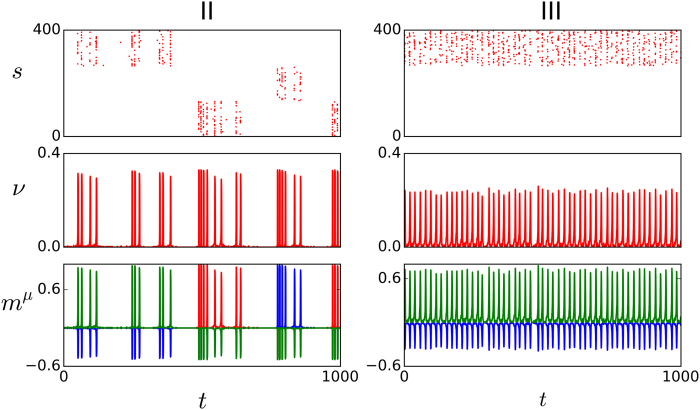
Time (ms) series in typical cases corresponding to phases II (left, with noise parameter *D *= 30 pA) and III (right, *D *= 50 pA) for *P *= 3 random patterns with *a *= 1/3. As indicated, the panels show, respectively from top to bottom for each phase, the local activity *s*(*t*) of the 400 neurons in the system (indicated with a dot if the neuron is active at that time and leaving unmarked otherwise), the *mean net activity v*(*t*) and the *overlap m*^*μ*^(t). In this case, changing colors indicate that the global activity is moving among the neighborhoods of the (three) memories. This is for *τ*_reco_ = 300 ms, *τ*_fac_ = 0, *d* = 0 and *I*_0_ = 25 pA.

Before we discuss our main results in the next section, it is convenient to describe qualitatively here the variety of phases at which the system stabilizes (generally after a transient time) depending on the noise intensity. This phenomenology was essentially reported before^19^,^51^, but there are important peculiarities and some new facts. As outlined in Fig. 2, one may distinguish seven qualitatively different cases as follows:

(I) For sufficiently small values of *I*_0_ (the parameter which in a way fixes the level of excitability in the system) and relatively low noise (e.g., this was observed for *D* = 20 pA), there is no activity and both *v*(*t*) and the overlaps *m*^*μ*^(*t*) are also constantly zero because noise is not enough to overcome the neuron thresholds. Any weak external signal is then muffled, given the absence of firing neurons capable of correlating with it. It is remarkable that this behavior is due to the underlying synaptic depression. In the absence of this, the activity would go to an attractor, but this is destabilized and quiescence sets in when synapses fluctuate. Something else is required to have then non-trivial global activity.

(II) The situation changes dramatically as the noise which results from the action of other regions is somewhat increased, e.g., for *D* = 30 pA. One then observes—but only for enough strength of synaptic fluctuations—a non-equilibrium phase with intermittent aperiodic activity in which bursts of firing neurons alternate with periods of silence, as in the left top panel of Fig. 3.

**Figure 4:**
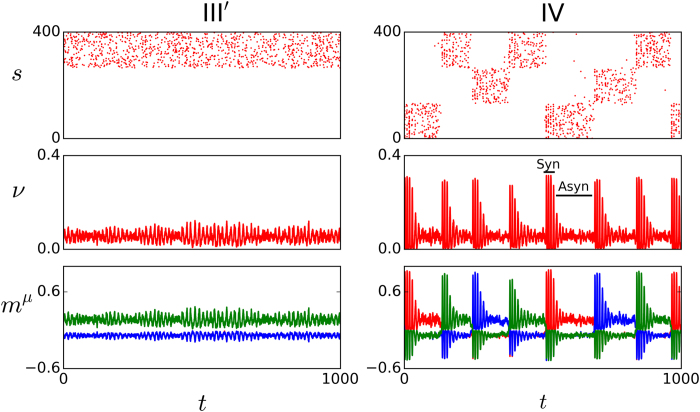
Same as in Fig. 3 but for III’ (left, *D *= 100 pA) and IV (right, *D *= 150 pA).

**Figure 5:**
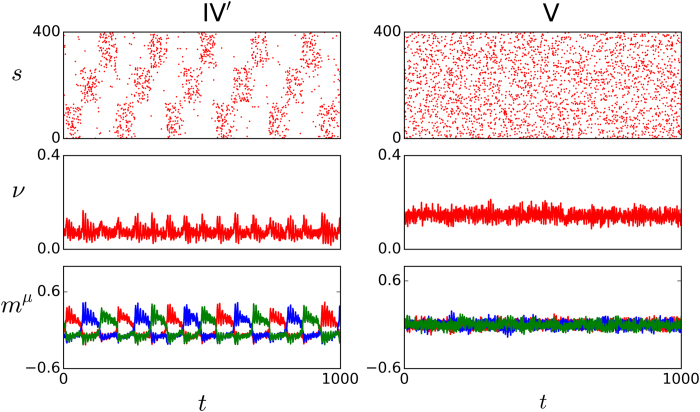
Same as in Fig. 3 but for IV’ (left, *D *= 300 pA) and V (right, *D *= 600 pA).

The neurons within each burst are strongly synchronized and belong to one of the patterns (three in this example) “stored” in the synaptic intensities *J*_*ij*_. This means that, following a completely unpredictable dynamics, the global activity moves from one pattern to another one—in fact, the pattern is never reached, given that the overlap *m*^*v*^ stays below unity, so that the move is between patterns neighborhoods. Eventually during this random roaming, the activity falls into a quiet state. One may describe this as a dynamic phase which consists of jumping between various metastable states—of which there are four in this case, the three memories and the quiet state. The roaming is quite irregular, as revealed in the bottom panel by changing colors, showing a tendency to visit evenly all the (in fact, equivalent) patterns. This non-equilibrium phase, which somehow mimics a condition of wakefulness with great attention, had not been described with this detail before (to the best of our knowledge).

(III) The silence time periods and the frequency of jumps between attractors, both tend to decrease as *D* is further (slightly) increased at constant excitability *I*_0._ Then, as in the right panels in Fig. 3, practically no silences occur and the activity gets finally clamped to one of the attractors, very much as in the familiar, equilibrium memory phase^50^. All the neurons which are active in that pattern are now firing periodically in a continuous series of synchronized population bursts.

(III’) As *D* is further increased for not too high excitability values, one may observe the situation which is illustrated by the left panels in Fig. 4. This is also a memory phase, but the involved neurons are now firing, due to the extra noise, in a random asynchronous manner, instead of synchronized which is the main feature of phase III.

(IV) Even larger values of *D* at low excitability tend to destabilize III’ (as well as III in the case that *I*_0_ is larger). A qualitatively new dynamic behavior as in the right panels of Fig. 4 then emerges. This consists of series of random jumps between metastable activity states in which the firing neurons belong to one of the stored patterns, i.e., unlike for case II, the quiet state is now unstable. Furthermore, in contrast with cases II and III, the degree of synchronization while in a metastable state now changes with time. That is, according to the indications in the second panel to the right of Fig. 4, the state starts with all the involved neurons firing synchronically in a very short time window, while it ends in an almost asynchronous activity state in which active neurons in the pattern fire at different times. There is kind of damped synchronization, which also echoes in the roaming among memories. Given that the attractor is characterized by all the active neurons firing at the same time, one concludes that the metastable states loose stability due to such progressive lack of synchrony, which is caused by the underlying noise *D* summing up to the synaptic depression which produces fatigue in the excitatory synapses after some time. The fatigue impedes, for instance, that a postsynaptic neuron fires immediately in response to a firing presynaptic neuron, which induces asynchrony. Both the degree of synchrony at the beginning of the metastable state and the life time of this state tend to decrease as *D* is increased. It is likely this kind of interesting complex behavior in some way is also reflected in actual brains.

(IV′) There is a value of *D* at which the metastable states become almost totally asynchronous since their start. The left panels in Fig. 5 illustrate this new condition. Note that, instead of the essential irregularity in case IV (which is obvious in the behavior of both *s*(t) and *m*^*v*^(*t*) in the right panels of Fig. 4), some periodicity now emerges spontaneously (there is no signal in the case illustrated here) which produces a maximum of correlation, as we shall see later on. The changing color of the overlaps in this case suggest periodic approaches to the neighborhoods of the stored patterns.

(V) The life times of the metastable states decrease continuously to zero, while the frequency of jumps increases, as *D* increases, and one finally reaches a narrow region around a critical value *D*_*c*_ where a global asynchronous state emerges. Neurons cannot fire now in synchrony due to the high level of noise, so that there is no correlation with any of the stored patterns (right panels in Fig. 5). This is a completely disordered, non-memory phase.

**Figure 6:**
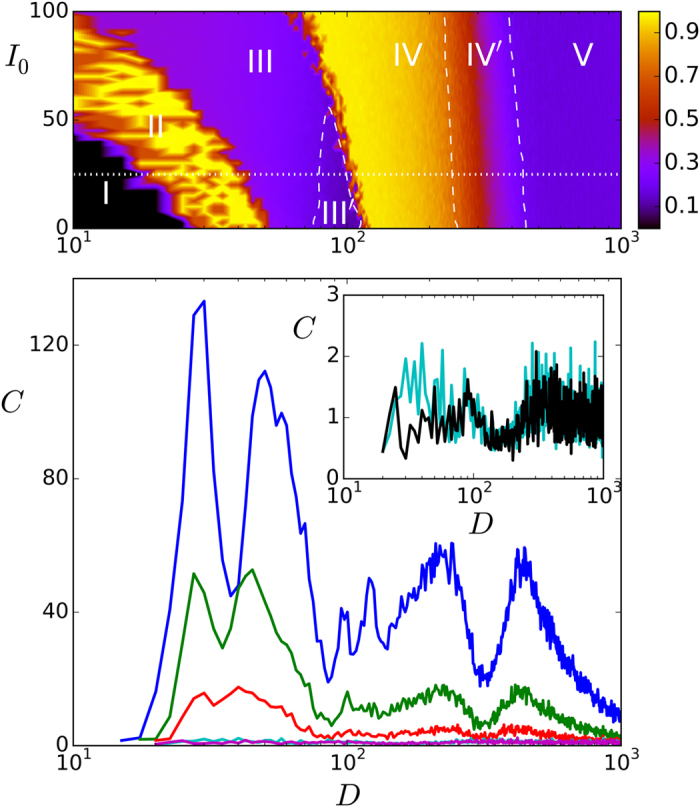
The lower panel shows plots of the variation with *D* (pA) of the input/output correlation *C* for different intensities *d* of the (weak) input signal. The main graph in this panel is, from top to bottom, for *d* = 20, 10, 5, 1 and 0.1. The last two ones are hardly to distinguish in this scale, and we show in the inset the same for *d* = 0 (black) and 0.1 (blue). The upper panel shows, for *d* = 20 pA, with different colors as indicated, how the (normalized) sum of the maximum values of all the overlaps varies with *D.* The parameters used here are the ones in Fig. 2, which shows a schematic representation of the upper panel here but in the absence of signal, i.e., for *d* = 0. These plots are based on 30 series of independent MC simulations involving 400 neurons and 6 memories (including negative ones).

Note that the horizontal axis in Fig. 2 is logarithmic so that the regions have a relatively much larger extension as one moves to the right, that is, IV and V correspond to a wide range of *D* while the regions holding the phases I and II are rather narrow. Also noticeable is that, even though somewhat hidden by finite-size effects in actual simulations, close inspection of series such as the ones in Figs 3, 4, 5 clearly indicates that the transitions I→II→III→IV are rather sharp, showing rapid changes of qualitative behavior, thus resembling the situation which is familiar from (equilibrium) discontinuous, first-order phase transitions. On the contrary, the transition IV→V is smoother, as it is also suggested by a rather continuous change of color in the top panel in Fig. 6, and it rather resembles a continuous, second-order phase transition instead. Further remarkable, on the other hand, is that the dashed lines in Fig. 2 do not locate modifications of the main global order but of the synchronization prototype. That is, the memory phase changes from synchronous to asynchronous as III→III’ and the synchronization degree decreases abruptly while roaming as IV→IV’. In any case, these transitions correspond to certainly significant changes of behavior which are clearly revealed by our “order parameter” *C*(*D*) so that one should probably term them also as (non-equilibrium) orthodox phase transitions^30^.

## Discussion

The qualitative phenomenology described above interests in relation with brain behavior. This biologically motivated model shows memory phases, extension of synchronization throughout the system, and quite irregular dynamics in which, e.g., several more-or-less-correlated attractors are visited in a way that mimics, say, *states of attention*^13^. In addition, narrow regions in parameter space describe qualitative changes bearing a close, essential similarity with familiar phenomena such as condensation and ferromagnetism. Furthermore, the model illustrates how a structured weak signal is able to survive competing with relatively strong random noise, that is, the system allows in some regions of its parameter space for an excellent propagation of weak signals, as illustrated in Fig. 6.

This figure shows typical cases of the function *C*(*D*). A first observation here is that the input/output correlation has “anomalous” increases, i.e., there is good propagation of the weak signal, precisely around the values of *D* for which we identified above relevant changeovers. This behavior is characteristic of *excitable media*, and it was previously described as “stochastic resonances”^19^. There is no need to call here for new phenomena, however, given that it seems already present for *d* = 0 (cf. Figs 2 and 6) (this is made even more explicit when one monitors directly, although not shown here, 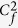 or 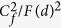, with *F* a smooth sigmoid function, which exhibits the same peaks for *d* = 0 than for nonzero *d*). That is, the six peaks in Fig. 6 precisely reflect the occurrence of the phase transitions I→II→III→III’→IV→IV’→V described above. This fact in a sense characterizes *C*(*D*) as the *order parameter* or perhaps the *susceptibility* for these nonequilibrium phase transitions. It follows the important result that monitoring appropriate quantities, namely, input/output correlations, e.g., in psychotechnic experiments concerning the transmission of weak signals competing with noise^19^,^51^,^52^, one may locate high level brain functions, including associated critical conditions. Note that such a task will in practice be eased by the fact that the peaks substantially increase with *d*, while the phase diagram remains practically unchanged (cf. the cases in Figs 2 and 6). Even though we anticipate that the analogy between brain properties and (non-equilibrium) phase transitions will certainly may be more involved in practice than suggested by our model, one should expect that analysis of such type of experiments will provide significant and useful information.

The model above may in fact be easily complemented along non-trivial lines, and the same picture is still supported. In addition to the related comments above, we mention that varying the depression and facilitation degrees in a comparable set up^53^ indicates so. In fact, if we move in the model the depression parameter around *τ*_reco_ = 300 ms in the absence of facilitation (*τ*_fac_ = 0) the location of the peaks along the *D* axis is moved in a similar way and percentage, and changing *τ*_fac_ from 100 to 500 ms (with *τ*_reco_ = 300 ms) just squeezes together the curves in graphs like the one in Fig. 6 and shifts them towards smaller *D*, but no qualitative changes occur. The only qualitative change is when one suppresses both depression and facilitation. In this case, the only phase transition is the equilibrium one from a memory phase to a completely disordered state^50^, and none of the above occurs.

We also checked that the behavior we are describing is quite robust if one increases the size *N* of the network; even a factor of five leaves unchanged both the peak locations and the width of the crossover regions. The number *P* of stored patterns is neither relevant as far as it is maintained small, though sufficiently increasing *P* has the effect of homogenizing the synaptic intensities, which is known to tend to trivialize the system behavior^50^. And modifying the nature of the neurons does not seem to induce important changes neither. For instance, assuming binary neurons—even though they lack the feeble and rapid, subthreshold oscillations of *integrate-and-fire* neurons before the potential *V* fires (Fig. 1)—has the only effect, as expected of obscuring the synchronization changes III→III’ and IV→IV′.

More intriguing is, in principle, the possible influence on emergent phenomena of the network topology, i.e., the adjacency matrix *ε*_*ij*_. Our graphs above are for fully connected networks, which is not realistic except for describing a very high level of connectivity, but other networks have also been investigated rather systematically^53^ and no essential changes ensue. In fact, we checked the behavior of the present model when a fraction, even large, of links is randomly suppressed in a fully connected network. The same peaks occur but with their location shifted towards lower levels of noise as dilution increases. This surely reflects a tendency of the attractors to loose stability so that the memory phase may even disappear for high dilution. We also simulated network topologies that are scale free, with different power-law distributions of connectivity, and some even having the small-world property as in the Watts-Strogatz case^53^. As expected, the only qualitative changes in the resulting behavior occur when long range connectivity is suppressed.

**Figure 7:**
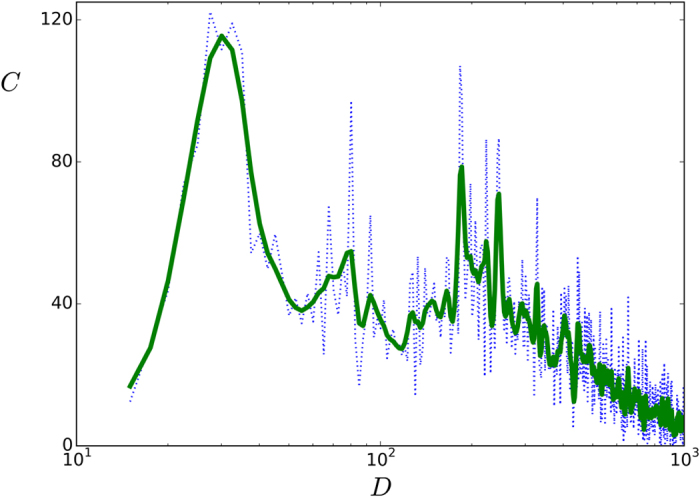
The function *C*(*D*) as in Fig. 6 when the adjacency matrix *ε*_*ij*_ corresponds to the Homo sapiens connectome. As given by an average of (undirected, symmetric) data from five cases, each with around 1000 nodes, including a coarse graining to 66 nodes^54^. The dotted line joins the data points. The solid (green) line is a guide to the eye obtained from a cubic spline. (The computer simulation here is for *d* = 40 pA and other parameters as before).

In order to complete the consideration of different topologies, and to further highlight the potential utility of the above model, we also implemented the adjacency matrix *ε*_*ij*_ with actual data^54^. Note that these data is “rough” within the context of our model given that it concerns interregional cortical connectivity and not even *canonical microcircuits* which would more closely correspond to the “synaptic connections” in our model. However, lacking more fine representation of the synaptic wiring it is interesting to check the consequences of using this net within the present context. The result is illustrated in Fig. 7 which is to be compared with the curves in the lower panel in Fig. 6. That is, using this description of connections in actual brains, the result is qualitatively similar to the one for the fully connected case.

We therefore conclude that our main point here, in addition to consistent with (rough) data, is independent of the network structure. One may understand this interesting fact as associated with the presence of factors *J*_*ij*_*x*_*j*_ in eq. (3). Such dynamic contributions already make the synaptic currents 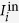 rather complex, even if networks were homogeneous. This would explain that it is often difficult to distinguish in practice between brain functional and structural complexity^55^,^56^. It also follows again that the dynamic complexity of synapses is at the origin of a varied, bizarre phenomenology and essential in determining the brain performance. No doubt that transmission experiments such as the ones mentioned above would greatly help in clarifying questions here raised.

## Additional Information

**How to cite this article**: Torres, J. J. and Marro, J. Brain Performance *versus* Phase Transitions. *Sci. Rep.* **5**, 12216; doi: 10.1038/srep12216 (2015).
